# A rapid 3% polyacrylamide slab gel electrophoresis method for high through put screening of LDL phenotype

**DOI:** 10.1186/1476-511X-7-47

**Published:** 2008-11-26

**Authors:** Yogendra Singh, Ramakrishnan Lakshmy, Ruby Gupta, Vemparala Kranthi

**Affiliations:** 1 Department of Cardiac Biochemistry, All India Institute of Medical Sciences, Ansari nagar, New Delhi-110029, India

## Abstract

**Background:**

Small dense LDL is reported to be associated with increased coronary artery disease risk by various epidemiological studies. The gold standard for separation and identification of LDL subtypes in plasma is ultracentrifugation which is a lengthy procedure and difficult to perform. Various other methods like NMR, HPLC, gradient gel electrophoresis (GGE) have been reported for LDL sub fractionation all of which require specialized equipments and expertise. We report here a high throughput 3% polyacrylamide slab gel electrophoresis method (PASGE) for sub fractionation of LDL which was compared with GGE, a commonly used method for LDL sub fractionation.

**Results:**

The 3% PASGE method compared well with the GGE method There was a good correlation between LDL particle diameter identified by the PASGE and GGE (Pearson correlation coefficient = 0.950). A 100% concordance was found when samples were classified as per LDL phenotypes in subjects with A and B phenotype by the two methods with the concordance being 66% in subjects with intermediate (I) phenotype. The electrophoresis apparatus was optimized and designed for running twenty eight samples at a time compared to twelve to fourteen by the conventional PASGE and eight to twelve by disc electrophoresis.

**Conclusion:**

The rapid 3% polyacrylamide slab gel electrphoresis method developed is simple to perform, cost-effective and can be used for the identification LDL sub fractionation and phenotyping in large epidemiological studies.

## Background

Cardiovascular disease (CVD) is the major public health problem in developing countries like India [1,2]. More alarming is the fact that it occurs a decade earlier in Indians compared to their western counter parts. In the Indian population, incidence of pre mature coronary artery disease (CAD) is high as compared to in western communities [3]. Metabolic syndrome characterized by truncal obesity, insulin resistance, non-insulin dependant diabetes, glucose intolerance, hypertension and atherogenic dyslipidemia is considered as the major predisposing factor for cardiovascular disease (CVD) in Indians. One of the important components of atherogenic dyslipidemia is small, dense low density lipoproteins (sd LDL).

Serum LDL has been the subject of numerous epidemiological studies because of their unequivocal association with coronary artery disease [4]. However nearly one third of patients with premature CAD have normal LDL cholesterol levels suggesting the fact that LDL sub fractions may be more important determinants for the predisposition to CAD. LDL particles are heterogeneous in nature differing in density, size, lipid composition, electrical charge, and pathological properties [5]. Small dense LDL particles readily undergo oxidative modification, which is an important step in the development of the arterial fatty streaks that lead to atherosclerosis, and also strongly associated with premature CAD [6]. Studies have reported an increase in coronary heart disease risk associated with presence of small, dense LDL [7-9].

Several methods are available for sub fractionation of LDL. These methods include density gradient ultracentrifugation [10], non-denaturing gradient gel electrophoresis (GGE) [11,12], nuclear magnetic resonance spectroscopy (NMR) [13] and High-performance liquid chromatography [14]. All these methods require specialized equipments which are not available in clinical chemistry laboratories. They are labor intensive, time consuming, expensive and not suitable for high-throughput screening. Commercial systems like the Quantimatrix Lipoprint System [15] (Dade Behring, USA) and Liphophor gel [16] (Jako) take relatively shorter time but are very expensive. Thus, there is need for a method for the LDL sub fractionation that would be simple, less expensive and thereby amenable to wide spread use. Here we are reporting a simple native polyacrylamide slab gel electrophoresis method which is time saving, needs no specialized equipments and would be suitable for screening of large number of samples. The method was compared against GGE which is extensively used for LDL sub fractionation.

## Results

**Figure 1:**
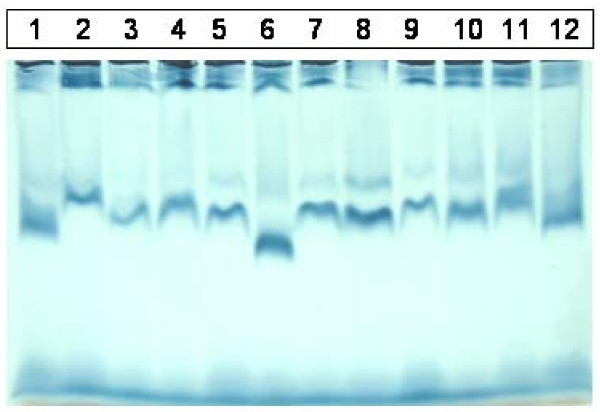
Plasma samples run on by 2 – 8% GGE.

**Figure 2:**
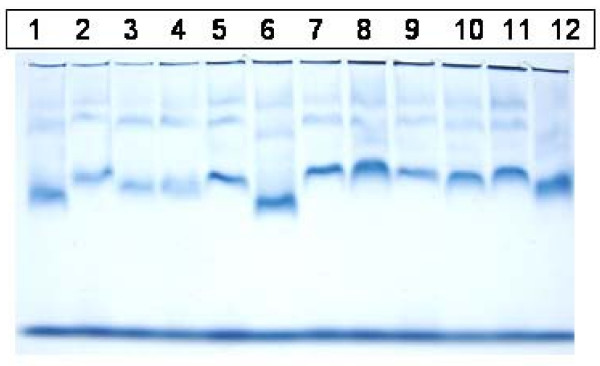
Plasma samples run on 3% PASGE.

**Figure 3:**
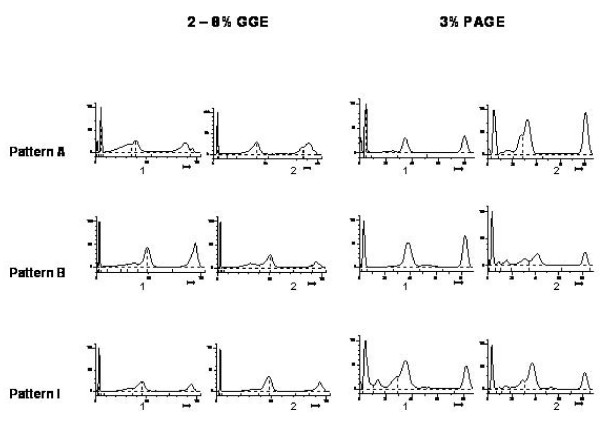
Densitometric pattern of samples run on GGE and 3% PASGE.

**Figure 4:**
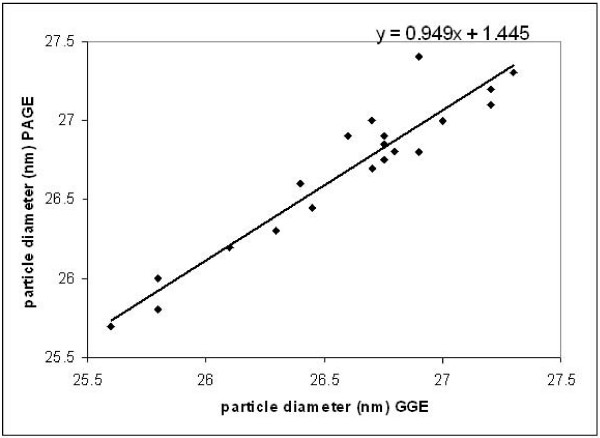
Relationship between LDL particle diameters obtained with 3% PASGE and GGE.

**Figure 5:**
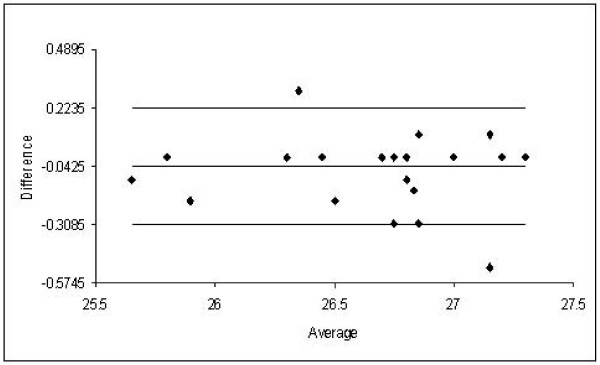
Bland Altman Plot of difference between LDL particle diameters obtained with 3% PASGE and GGE.

Twenty samples were analyzed by both 3% PASGE method and GGE. Gel pictures of LDL fractionation of 12 samples by 2–8% GGE and 3% PASGE is depicted in Fig 1 &2 respectively. As is evident a good separation of LDL fractions was achieved by both the methods. The densitometry pattern of three samples of different LDL phenotypes identified by the two methods is depicted in Figure 3. The particle diameter was arrived at from known standards run along with the unknown samples. Inter assay coefficient of variation for 3% PASGE was arrived at by running the two samples in three different runs and was 0.93% (mean diameter 26.39 nm) and 0.71% (mean diameter 26.78 nm). The intra assay CV was obtained by running two samples in triplicates in the same run and was 0.75% (mean diameter 24.4 nm) and 1.19% (mean diameter 25.58 nm). A good correlation was evident between particle diameter identified by the PASGE as compared to GGE (Fig 4, Pearson correlation coefficient = 0.950). The Bland Altman plot of difference in particle diameter between GGE and PASGE plotted against particle diameter (Fig 5) showed a good agreement between the two methods with 18 out of 20 samples falling within the 95% confidence interval. A 100% concordance was found when samples were classified as per LDL phenotypes (diameter < 25.8 as B phenotype, 25.8–26.3 as Intermediate phenotype (I) and > 26.3 as A phenotype) in subjects with A and B phenotype. 3 samples were identified as having "I" phenotype by 3% PASGE of which one was identified as having pattern B by 2–8% GGE (concordance 66%, Kappa statistics 0.67).

## Discussion

Different methods have been reported for separation of LDL fractions and include NMR, HPLC, ultracentrifugation and GGE. The first three require sophisticated instruments which may not be available in all clinical chemistry laboratories. LDL sub-fractionation by GGE has been widely used by clinical laboratories [7-9] however the method is time consuming (takes 12–14 hours). Also, high-quality non-denaturing gradient gels are difficult to prepare [17]. Commercial systems like the quantimatrix lipoprint system (Dade Behring, USA) and liphophor gel (Jako) are available for LDL subfractionation and have been reported in number of studies [18-21] but are very expensive therefore are not viable for routine testing. Both these commercial methods use tube gel for LDL separation and therefore only 8 to12 samples can be analyzed at a time. Hirano et al [18] compared polyacrylamide tube gel electrophoresis method performed on commercially available lipoprint system with GGE. The authors reported a good agreement between the two methods with a weighted Kappa of 0.78. The 3% slab gel method described here for separation of LDL takes approximately 3 hrs and is cost effective. The method compared well with GGE with a concordance of 100% for A and B phenotypes and 66% for intermediate phenotype. To increase the throughput we employed a dual electrophoresis system on which 28 samples could be loaded at one time. This is much higher than tube gel where only 8 to 12 samples can be analyzed in one run or single slab gel on which 12–14 samples can be loaded at a time. This is particularly useful for epidemiological studies where large number of samples need to be analyzed.

## Conclusion

The 3% slab gel electrophoresis method described is simple, cost effective, takes shorter time and allows measurement of larger number of samples compared to other reported methods for LDL subfractionation.

## Methods

Blood samples were drawn after overnight fast from 20 individuals in Na2 EDTA vacutainers (Becton Dickson) and plasma was separated by centrifugation at 2000 rpm. Samples were aliquoted and stored at -70°C until LDL sub fraction analysis by 3% PASGE and Gradient gel electrophoresis (GGE).

### Polyacrylamide slab gel electrophoresis

A 3% PASGE method was developed by modification of existing electrophoresis methods for LDL sub fractionation [11,15,22]. Acrylamide, bis-acrylamide, TEMED and APS were of analytical grade and were purchased from Sigma – Aldrich. Stock solutions used were 29% acrylamide (solution 1) and 1% bis acrylamide (solution 2). 3% slab gel was prepared by mixing 3.7 ml of solution 1, 3.7 ml of solution 2, 0.1 ml of TEMED and 0.5 ml of APS. The gels were allowed to polymerize overnight in cold and used within 2 days of preparation. Plasma samples were pre stained with Sudan black B by mixing 25 μl of plasma with 20 μl of 1% (w/v) Sudan black B. The samples were allowed to stain overnight. 40 μl of pre stained sample were loaded into the wells. Pre stained carboxylated polystyrene beads (diameter 40.0 nm), apoferritin (diameter 12.2 nm) and thyroglobulin (diameter 17.0 nm) were used as standards. Standard proteins were stained after electrophoresis in Coomassie brilliant blue (0.25 g in 45 ml methanol, 10 ml glacial acetic acid) for 6 hrs, and then de-stained using 50% methanol and 40% glacial acetic acid. Samples of known LDL diameter were incorporated in each run for quality assurance.

Electrophoresis was performed in cold (4–8°C) using TBE buffer (90 mM tris base, 80 mM boric acid, and 3 mM EDTA, pH 8.3). The gel was pre run for 10 minutes at 50 V. Samples were run initially at 70 V for 30 min, followed by 125 V for 1 hr and 200 V for 1.5 hrs. Gel was allowed to remain in dark for 1 hour. Densitometry was performed in a Helena EDC system (Helena laboratories).

The migration of LDL and HDL was arrived at by measuring the distance between absorbance maxima of VLDL and LDL and that of VLDL and HDL respectively. Migration of predominant LDL fraction in relation to HDL was derived as follows

$$\text{Relative migration of LDL}=\frac{\text{Distance between VLDL and LDL band}}{\text{Distance between VLDL and HDL band}}$$

Particle diameter corresponding to LDL peaks was calculated from a calibration curve prepared from standards of known diameters which were incorporated in every run.

### Gradient Gel Electrophoresis

For comparison, samples were run on non denaturing 2–8% polyacrylamide gradient gel. LDL subfractionation by GGE [16] was carried out at 4–8°C at 125 V for 12 – 14 hrs using TBE buffer (90 mM Tris HCl, 80 mM boric acid, and 3 mM EDTA, pH 8.3). The gels were pre run at 25V for 10 min prior to the loading of pre stained samples. After the electrophoresis, the protein standards apoferrtin (12.2 nm) and thyroglobulin (17.0 nm) were stained in 0.25% coomassie brilliant blue solution followed by destaining for 6 hours. Densitometric scanning was performed as described above to measure the peak diameter of LDL sub fractions.

### Statistical methods

Agreement between PASGE and GGE method was evaluated by applying Pearson correlation. Bland Altman plot was generated to assess the deviation of PASGE method from GGE. Weighted Kappa statistics was applied to evaluate concordance between LDL phenotypes obtained by the two methods.

## Abbreviations used

CVD: Cardiovascular Disease; PASGE: Polyacrylamide Slab Gel Electrophoresis; GGE: Gradient Gel Electrophoresis; NMR: Nuclear Magnetic Resonance; LDL: Low: density lipoprotein; VLDL: Very low: density lipoprotein; TEMED: N, N, N', N': tetramethylene diamineAPS: Ammonium persulphate.

## Competing interests

The authors declare that they have no competing interests.

## Authors' contributions

YS was responsible for analyses of blood samples and initial drafting of the manuscript. RL was responsible for design, planning, execution and drafting of the manuscript, RG and VK were involved in drafting the manuscript and revising it critically for intellectual content.
